# Oral Candidiasis Mimicking Mucosal Lesions of Bullous Pemphigoid During Systemic Corticosteroid Therapy: A Case Report

**DOI:** 10.4317/jced.63038

**Published:** 2025-09-01

**Authors:** Jumi Nakata, Yoshihito Maruyama, Kana Ozasa, Andrew Young, Noboru Noma

**Affiliations:** 1 DDS, Department of Oral Medicine, Nihon University school of Dentistry Tokyo, Japan; 2 DDS, MSD Department of Diagnostic Sciences, Arthur Dugoni School of Dentistry, University of the Pacific, San Francisco, United States; 3 MD, Division of Internal Medicine, Towa Hospital, Tokyo 120-0003, Japan

## Abstract

Bullous pemphigoid (BP) is an autoimmune blistering disorder mainly affecting the elderly. Although primarily cutaneous, bullous pemphigoid can occasionally involve the oral mucosa, which complicates diagnosis and management.

We report an 88-year-old woman with diabetes and dementia who presented with tense bullae on her limbs. Systemic corticosteroid therapy improved the skin lesions, but new painful erosions appeared on the hard palate. Although mucosal BP was considered, the concurrent improvement of cutaneous symptoms suggested an opportunistic infection instead. Culture confirmed *Candida albicans*, and antifungal therapy led to rapid resolution. This case highlights the diagnostic challenge of distinguishing between BP progression and steroid-induced candidiasis. Early detection and proper treatment are essential, especially in elderly, immunocompromised patients. Maintaining oral hygiene through dental collaboration also plays a crucial role in preventing such complications. Our findings underscore the importance of multidisciplinary care and managing infection when treating BP in patients with systemic vulnerabilities.

** Key words:**Bullous pemphigoid, Oral candidiasis, Elderly patient with diabetes.

## Introduction

Bullous pemphigoid (BP) is an autoimmune subepidermal blistering disease characterized by IgG autoantibodies targeting hemidesmosomal proteins BP180 and BP230 in the epidermal basement membrane [1]. Clinically, it presents with tense bullae and pruritic edematous erythema, typically on the skin but occasionally involving the oral mucosa [2]. Diagnosis is based on clinical features, serological tests for BP antibodies, and histopathological findings.

Systemic corticosteroids remain the standard treatment, typically resulting in resolution of bullous lesions. In some cases, combination therapy with tetracycline and nicotinamide is also effective. However, systemic steroids may cause side effects such as moon face, gastrointestinal ulcers, and immunosuppression.

Herein, we report a case of BP in which systemic steroid therapy led to improvement of cutaneous lesions, yet erosions appeared in the oral cavity during the treatment, requiring differentiation between disease progression and a steroid-related opportunistic infection of oral candidiasis.

## Case Report

An 88-year-old woman residing in a nursing facility presented to our hospital in March 2024 with blisters on both palms, which subsequently spread to both lower legs; her medical history included diabetes mellitus and dementia (onset dates unknown), sigmoid colon cancer treated with low anterior resection in 2018, and a left femoral fracture managed with open reduction and internal fixation in 2022. At the time of presentation, the patient was taking glimepiride 1.0 mg twice daily, metformin 250 mg once daily, sennoside 24 mg once daily, famotidine 20 mg twice daily, and magnesium oxide 660 mg twice daily.

- Examination findings

**Figure 1:**
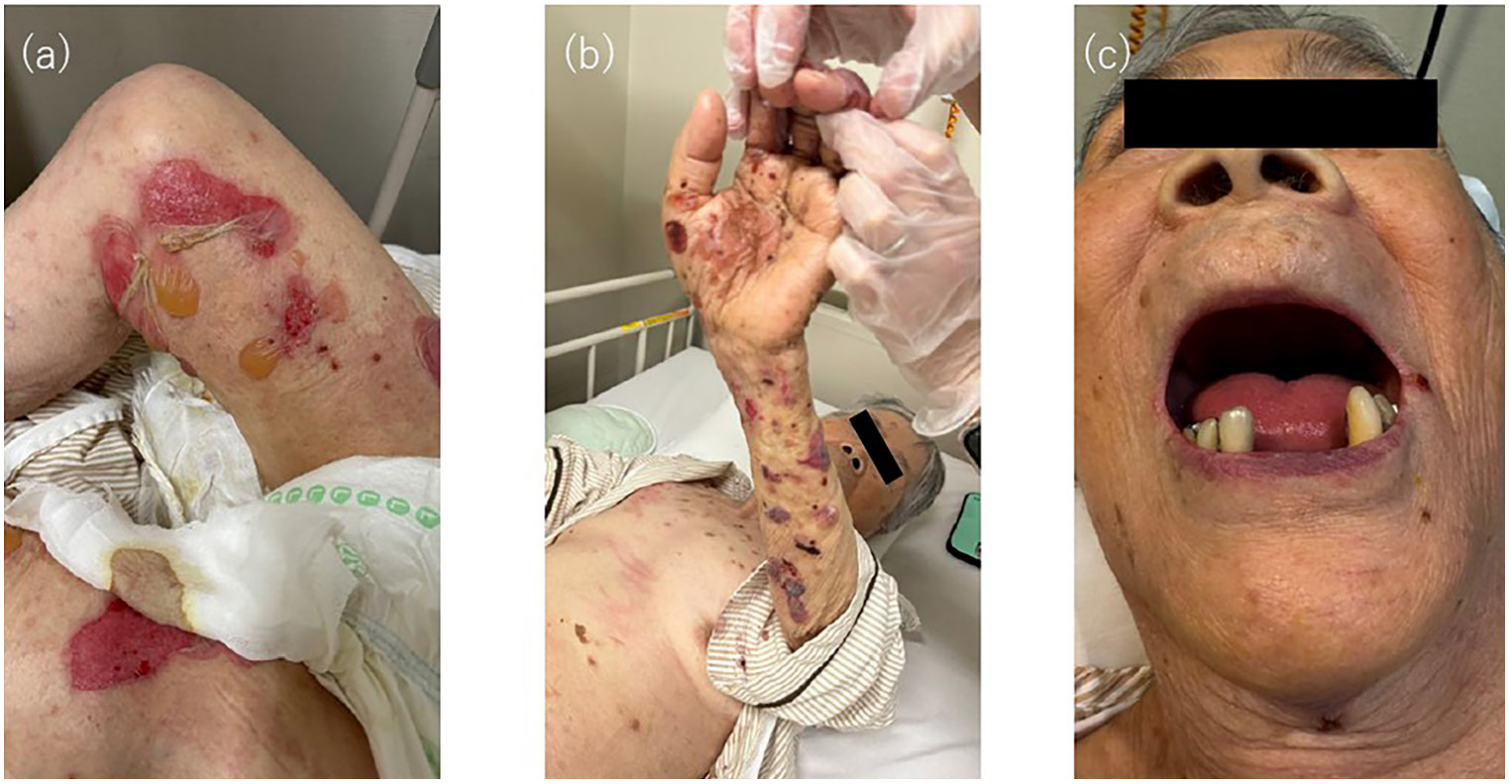
(a) Tense bullae with surrounding erythema on the lower legs at initial presentation. (b) Multiple tense bullae on the left palm. (c) Intraoral findings showing an edentulous maxilla and remaining mandibular anterior teeth with missing molars; no mucosal abnormalities were observed.

The dermatological examination revealed multiple tense bullae on both palms and lower legs, along with erythema and scratch marks on the trunk (Fig. 1a,b).

The oral examination showed an edentulous maxilla, and the remaining mandibular teeth limited to the anterior region with missing molars. No mucosal abnormalities were observed at the initial visit (Fig. 1c).

Laboratory findings were noTable for hypoalbuminemia (Alb 3.2 g/dL), hyponatremia (Na 129 mEq/L), mild leukocytosis (WBC 7,500/μL), elevated CRP (2.32 mg/dL), hyperglycemia (Glu 191 mg/dL), elevated HbA1c (7.7%), and a markedly elevated anti-BP180 antibody level of 493 U/mL.

Based on the characteristic skin lesions and serological findings, a diagnosis of BP was established.

The patient was admitted for close monitoring of her diabetes during systemic steroid therapy. Treatment was initiated with oral prednisolone 25 mg/day, minocycline 100 mg/day, and nicotinamide 200 mg/day, with a plan to taper prednisolone by 5 mg every two weeks.

**Figure 2:**
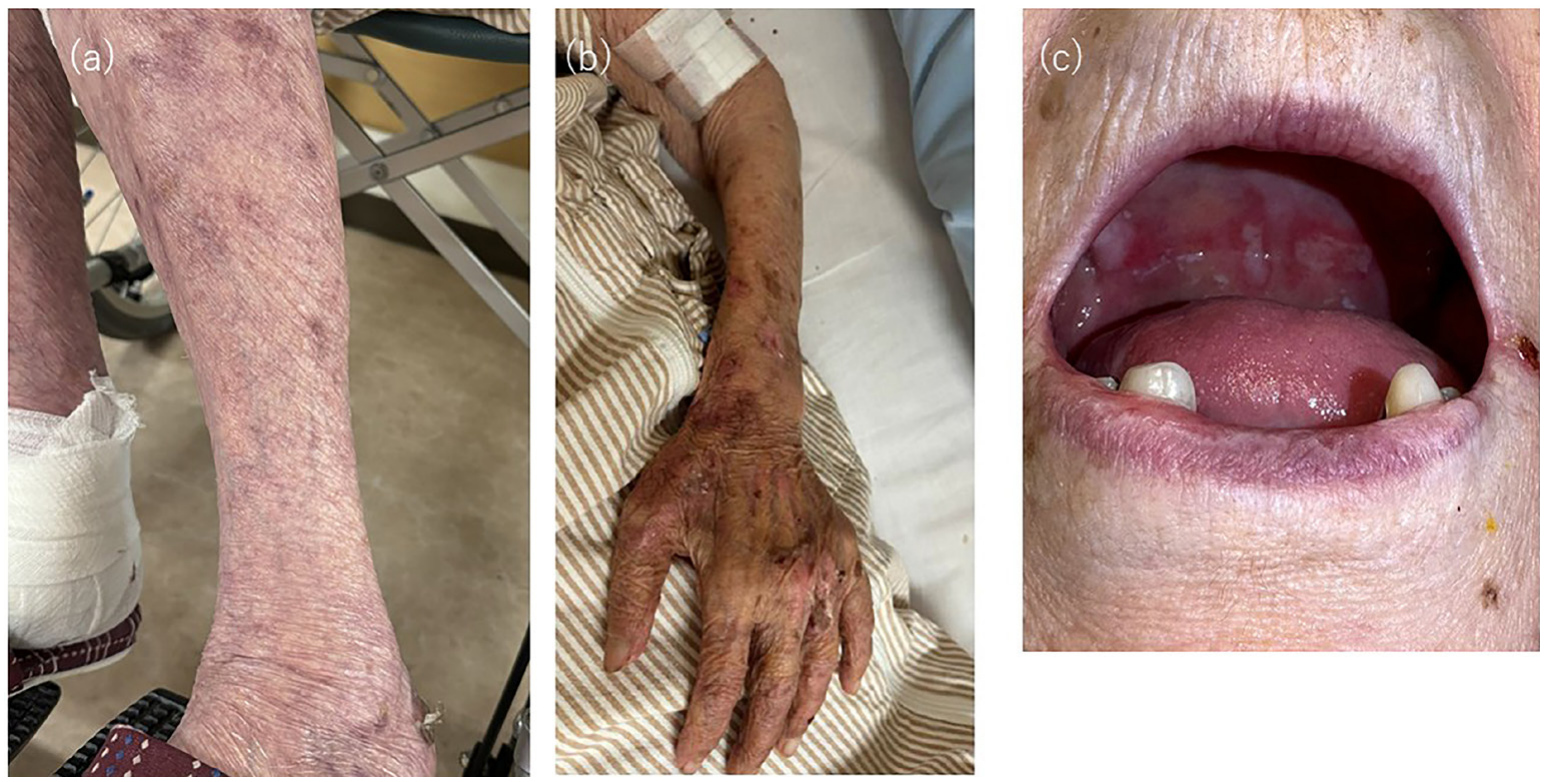
(a,b) By day 14 of treatment, most of the bullous lesions on the lower limbs had crusted and begun to heal. (c) Erosive lesions on the hard palate observed after the onset of oral pain, suggesting possible mucosal involvement.

Initially, the patient had severe pruritus and widespread blisters and erosions on the limbs. By day 14, most limb lesions had crusted and healed, and prednisolone was tapered to 20 mg/day (Fig. 2a,b). However, a few days later, she began complaining of oral pain during meals, which led to decreased oral intake. Examination revealed erosions on the hard palate (Fig. 2c). Although mucosal involvement of BP was suspected, the simultaneous improvement of cutaneous lesions raised the possibility of a secondary process.

**Figure 3:**
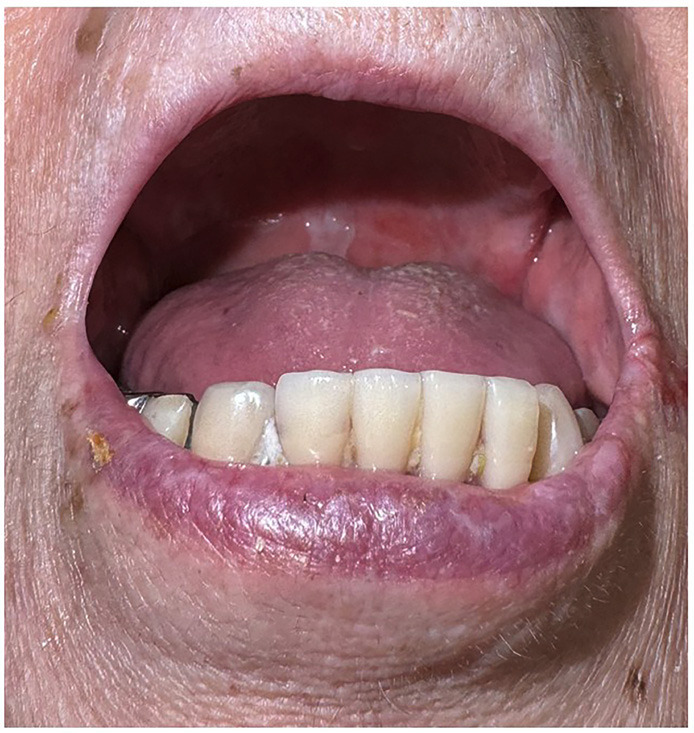
Improvement of oral erosions was observed after antifungal therapy.

Given her age, diabetes, and steroid therapy, opportunistic infection was suspected. Culture of the palatal erosions yielded *Candida albicans*, leading to a diagnosis of oral candidiasis. Treatment with miconazole oral gel 400 mg/day resulted in rapid resolution of lesions (Fig. 3).

## Discussion

We report a case of oral candidiasis clinically resembling a mucosal relapse of BP during systemic steroid therapy. Bullous pemphigoid typically presents with cutaneous lesions, while mucosal involvement, including the oral cavity, is relatively uncommon [1]. In the present case, the patient initially exhibited edematous erythema and large tense bullae, primarily on the extremities, accompanied by intense pruritus (Fig. 1a,b). Given the clinical findings and the positive result for anti-BP180 antibodies, a diagnosis of bullous pemphigoid was made. At that time, no lesions were observed in the oral cavity (Fig. 1c).

Following the diagnosis, systemic corticosteroid therapy was initiated, resulting in the resolution of the cutaneous bullae with residual pigmentation (Fig. 2a,b). However, despite the improvement in the skin lesions, multiple painful erosions developed on the hard palate (Fig. 2c). This presented a diagnostic challenge: whether the oral lesions represented a progression of bullous pemphigoid, or the coexistence of another condition such as oral candidiasis.

Oral lesions in autoimmune blistering diseases can be confused with inflammatory or infectious conditions. Misdiagnosis with candidiasis or herpetic infections has been documented in pemphigus vulgaris and BP [2]. Elderly and diabetic patients are particularly prone to opportunistic infections, especially under immunosuppressive therapy. In BP, systemic steroids and diabetes are independent risk factors for infection, with an odds ratio of 2.667 for diabetic patients [3]. Candidiasis commonly develops under such conditions, with an estimated 4.4-fold increased risk in diabetic individuals [4]. Hyperglycemia and immune suppression both facilitate fungal overgrowth. In this patient, oral erosions developed during steroid tapering, suggesting that host susceptibility due to age, diabetes, and immunosuppression played a pivotal role.

Clinically, distinguishing candidiasis from mucosal involvement of BP is challenging. *Candida* lesions may present as white pseudomembranes or erythematous erosions, with characteristic detachment on gentle wiping. In contrast, BP bullae are fluid-filled and non-pseudomembranous. However, both conditions may appear as erosions when lesions are ruptured or advanced. Pain and burning sensations tend to be more prominent in candidiasis than in BP. In this case, oral pain despite improvement in general condition hinted at an alternate etiology.

Initially, a biopsy of the lesion was considered for differential diagnosis. However, due to the patient’s advanced age and dementia, it was difficult to obtain consent and cooperation (such as mouth opening) for procedures. In addition, since the lesion was located on the hard palate, there was a risk of aspiration and potential airway obstruction if biopsy-related bleeding were to occur. Therefore, we decided to perform a culture and, if *Candida* was detected, initiate antifungal therapy to evaluate the treatment response. Based on the therapeutic outcome, we would then differentiate between oral candidiasis and other possible conditions, including BP. In this case, the lesion resolved promptly with antifungal treatment, suggesting that the oral lesion was not a recurrence of BP, but rather oral candidiasis. When clinical distinction is difficult, microbiological , in applicable cases, histopathological investigations should be promptly performed to guide treatment.

- Therapeutic Considerations

Initial treatment with prednisolone 25 mg/day was in line with guidelines recommending 0.5–1.0 mg/kg/day for moderate-to-severe BP. Steroid tapering over months is standard, transitioning to maintenance doses of 5–10 mg/day [5]. However, older patients are more vulnerable to steroid-related complications, and relapse during tapering is common. Approximately 30% of BP patients relapse within one year of remission, and up to 50% within several months of therapy cessation [6].

Steroid-sparing agents (methotrexate, azathioprine, mycophenolate mofetil) or advanced therapies (IVIG, plasma exchange, rituximab) may be considered in refractory cases. For mild disease, topical steroids or tetracycline-nicotinamide combinations are effective [7].

In our case, early recognition of *Candida* infection allowed a switch to antifungal therapy, avoiding further immunosuppression. This emphasizes the importance of differentiating between disease activity and treatment-related complications.

To prevent recurrence and optimize management, steroid tapering should consider glycemic control and overall condition. Monitoring for infections, particularly oral candidiasis, is essential. Japanese guidelines recommend antifungal gargles during steroid therapy, and oral care collaboration with dental professionals is vital.

Improved oral hygiene reduces risks of candidiasis and dental caries in autoimmune blistering diseases and enhances quality of life.

This case highlights the importance of managing infections and treatment-related side-effects in elderly patients with BP and comorbidities, to ensure safe and effective long-term disease control.

## Conclusions

The appearance of new oral erosions during treatment for BP posed a diagnostic challenge in differentiating between disease exacerbation and coexisting oral candidiasis. This case highlights the importance of early detection and management of infections, as well as the role of oral hygiene, in the management of BP in elderly patients with diabetes.
